# Prognostic Impact of Surgical Margin in Hepatectomy on Patients With Hepatocellular Carcinoma: A Meta-Analysis of Observational Studies

**DOI:** 10.3389/fsurg.2022.810479

**Published:** 2022-02-09

**Authors:** Yeting Lin, Jiaxuan Xu, Jiaze Hong, Yuexiu Si, Yujing He, Jinhang Zhang

**Affiliations:** ^1^Anesthesiology Department, Ningbo Yinzhou No. 2 Hospital, Ningbo, China; ^2^The Second Clinical Medical College, Zhejiang Chinese Medical University, Hangzhou, China; ^3^School of Basic Medical Sciences, Zhejiang Chinese Medical University, Hangzhou, China; ^4^Surgery Department, Fenghua Hospital of Traditional Chinese Medicine, Ningbo, China

**Keywords:** hepatocellular carcinoma, hepatectomy, surgical margin, prognosis, meta-analysis

## Abstract

**Objective:**

This study aims to comprehensively evaluate the prognostic impact of the surgical margin in hepatectomy on patients diagnosed with hepatocellular carcinoma (HCC).

**Methods:**

A comprehensive and systematic search for eligible articles published in English before July 2021 was conducted across PubMed, Cochrane Library, Web of Science, and Embase electronic databases. The overall survival (OS) and disease-free survival (DFS) were the primary endpoints.

**Results:**

In total, 37 observational studies with 12,295 cases were included in this meta-analysis. The results revealed that a wide surgical margin (≥1 cm) was associated with better OS (hazard ration (HR), 0.70; 95% confidence interval (CI), 0.63–0.77) and DFS (HR, 0.66; 95% CI, 0.61–0.71) compared to a narrow surgical margin (<1 cm). Subgroup analyses were conducted based on median follow-up time, gender, country, hepatitis B surface antigen (HBsAg) status, tumor number, and liver cirrhosis. The prognostic benefit of a wide surgical margin was consistent in most subgroups, however, analysis of studies from Western countries showed that margin width was not associated with prognosis.

**Conclusion:**

In summary, a surgical margin wider than 1 cm prolongs the long-term prognosis of HCC patients compared to a surgical margin narrower than 1 cm.

## Introduction

Although hepatocellular carcinoma (HCC) has the 5^th^ highest incidence across the globe, it is currently the 3^rd^ leading cause of cancer-related deaths (1, 2). So far, liver transplantation, hepatic resection and radiofrequency ablation are the few treatment strategies for HCC. Although hepatectomy is the first-line therapeutic intervention, the prognosis of patients is unsatisfactory due to the high risk of recurrence (70% in the 5^th^ year after surgery) and metastasis (3).

The long-term prognosis of patients with HCC is influenced by several factors, among them, liver cirrhosis is a main factor, and the surgical margin is considered a potential prognostic factor (4, 5). Curative hepatectomy is complete resection of all visible tumors without residual tumor cells at the resection margin (6). As such, an adequate resection margin is vital in preventing tumor recurrence (7). Nonetheless, minimizing the removal of the non-malignant parenchyma tissue and protecting the residual liver of liver resection is necessary for many HCC patients with liver cirrhosis or other liver diseases. This is because the capacity for liver regeneration is impaired among these patients and excessive liver tissue removal leads to severe consequences including liver failure (8, 9). Thus, controversies on the width of the surgical margin have been reported under the premise of R0 resection. Many studies reveal that the width of the resection margin narrower than 1 cm is a risk factor for the long-term prognosis of HCC patients after surgery (4, 10). Nevertheless, a number of articles found that a wide surgical margin did not improve the prognosis of HCC patients after hepatectomy (11, 12).

Therefore, this meta-analysis seeks to assess the correlation between surgical margins (a surgical margin wider than 1 cm; a surgical margin narrower than 1 cm) and long-term prognosis of HCC patients after hepatectomy.

## Methods

### Literature Search Strategy

This meta-analysis adhered to the guidelines from the Preferred Reporting Items for Systematic Review and Meta-Analysis (13). A comprehensive and systematic literature search for articles published in English before July 2021 was conducted in four online electronic databases including PubMed, Cochrane Library, Web of Science, and Embase. The search terminologies included: “Hepatocellular Carcinoma” OR “Liver Cell Carcinomas” OR “Hepatoma” OR “HCC” AND “Resection Margin” OR “Surgical Margin” OR “Margin Width.” Besides, reference lists of all retrieved papers were inspected to identify potentially eligible but uncaptured literature in the primary search.

### Inclusion Criteria

Studies were included if they met the following criteria: (1) The cancer type was primary HCC and hepatectomy was performed on patients; (2) Patients received different surgical margins in the study (a wide surgical margin, ≥1 cm) and control (a narrow surgical margin, <1 cm) groups; (3) The study was original, including retrospective and prospective observational studies (OBS); (4) Extractable outcomes were in the studies.

### Exclusion Criteria

The exclusion criteria for this meta-analysis included: (1) HCC was recurrent; (2) The patients received palliative hepatectomy or had extrahepatic metastases; (3) The study did not divide the study group and the control group into larger than 1 cm and smaller than 1 cm; (4) Duplicate article or repeat analyses using similar data.

### Data Extraction and Quality Evaluation

Data extracted from eligible studies included study characteristics (author, country, publication year, study design, median follow-up time, and mentioned outcome measures), demographic data of patients (age, gender, and the number of patients), and clinicopathological features (liver cirrhosis, virus status, tumor number and size, and serum alpha-fetoprotein (AFP), and survival outcomes.

The quality of incorporated OBSs was evaluated using the Newcastle-Ottawa Scale (NOS) based on three aspects i.e., patient selection, comparability of groups, and outcome evaluation. The scores of papers >6 were considered high-quality.

### Statistical Analysis

To evaluate the relationship between surgical margins and HCC prognosis, the overall survival (OS) and disease-free survival (DFS) in the wide surgical margin group vs. the narrow surgical margin group was compared using a pooled hazard ratio (HR) with its corresponding 95% confidence interval (CI). The degree of heterogeneity across included literature was assessed using the I^2^ statistic. Considering the potential heterogeneity, random-effect model was applied to all analyses. To assess the robustness of conclusions, a sensitivity analysis was conducted. *P* < 0.05 was considered statistically significant.

## Results

### Data Collection and Characteristics

**Figure 1:**
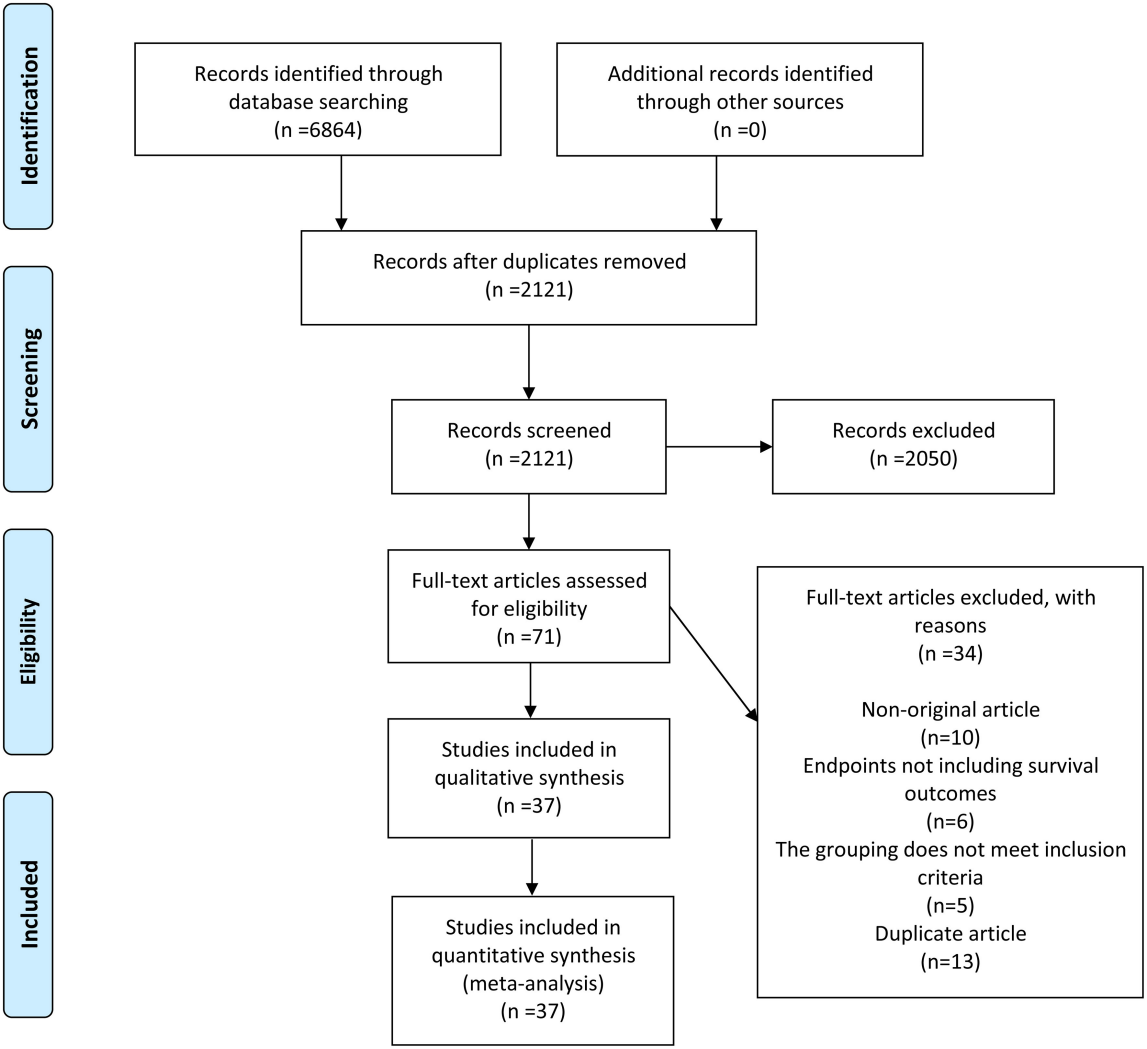
A schematic flow for selecting the articles included in the meta-analysis.

A total of 6,864 records were initially identified by the literature search. Out of these, 4,743 records were excluded because of duplication, and 2,050 records were eliminated after evaluating their titles or abstracts. The remaining 71 records were carefully inspected by full-text reading. Finally, 37 articles (4, 5, 7, 10–12, 14–44) were included. The comprehensive search and selection process is shown in (Figure 1).

The comprehensive characteristics of the included studies are summarized in (Table 1-1). The included articles were published between 1993 and 2021. A total of 12,295 patients from Western and Asian countries were enrolled in 37 OBSs; two studies of these were prospective, while the rest were retrospective. The majority of articles were from Asia, with China representing the most (24 articles). The demographic and clinicopathological characteristics of patients are presented in (Table 1-2). Based on a qualitative assessment by NOS criteria, the results revealed that all included OBSs were of higher quality (Supplementary Table 1).

**Table 1-1:** Characteristics of all the studies included in the meta-analysis.

**Author**	**Year**	**Country**	**Number of patients**		**Median follow-up (months)**	**Study design**	**Survival outcomes**
			**Wide resection margin (>1 cm)**	**Narrow resection margin (<1 cm)**			
Belli	2011	Italy	56	9	29.0	Retrospective	DFS
Chang	2012	China	478		29.5	Retrospective	DFS
Chen	2003	China	174	68	11.8	Retrospective	OS
Chen	2015	China	114	82	NA	Retrospective	OS
Chen	2021	China	176	238	>60.0	Retrospective	OS
Dong	2016	China	351	235	46.8	Retrospective	DFS
Han	2019	China	302	147	56.3	Retrospective	OS, DFS
Hirokawa	2014	Japan	10	10	46.0	Retrospective	DFS
Hsiao	2017	China	154	67	NA	Retrospective	OS
Huang	2013	China	528	512	42.0	Retrospective	OS, DFS
Huang	2015	China	71	159	72.0	Retrospective	OS, DFS
Laurent	2005	France	61	41	23.0	Retrospective	OS, DFS
Lee	1996	China	38	10	>60.0	Retrospective	OS
Lee	2007	Korea	44	56	31.0	Retrospective	OS, DFS
Lee	2012	China	142	156	73.0	Retrospective	OS, DFS
Lee	2018	Korea	186	233	37.5	Retrospective	OS, DFS
Lee	2019	China	143	391	66.3	Retrospective	OS, DFS
Lise	1998	Italy	72	15	29.0	Retrospective	OS, DFS
Liu	2016	China	186	37	26.1	Retrospective	DFS
Liu	2020	China	134	106	55.2	Retrospective	OS, DFS
Park	2018	Korea	61	31	28.0	Retrospective	OS, DFS
Poon	2000	China	138	150	27.0	Prospective	OS, DFS
Sasaki	2006	Japan	176	241	>120.0	Retrospective	DFS
Shi	2019	China	177	99	44.0	Retrospective	OS, DFS
Shimada	2008	Japan	32	85	62.0	Retrospective	OS
Shin	2018	Korea	55	61	66.7	Retrospective	DFS
Su	2021	China	45	114	61.2	Retrospective	OS, DFS
Takano	2000	Japan	244	56	NA	Retrospective	OS
Torii	1993	Japan	25	34	25.0	Retrospective	OS
Tsilimigras	2020	Multicenter	78	326	28.5	Retrospective	OS, DFS
Wang	2010	China	404	34	21.0	Retrospective	OS
Yang	2014	China	126	959	NA	Retrospective	OS, DFS
Zeng	2020	China	155	544	NA	Retrospective	OS, DFS
Zhang	2014	China	216	86	26.0	Prospective	DFS
Zhang	2021	China	305	120	26.0	Retrospective	DFS
Zhou	2020	China	92	217	NA	Retrospective	OS, DFS
Zhou	2021	China	325	492	NA	Retrospective	OS

*OS, overall survival; DFS, disease-free survival; NA, not available*.

**Table 1-2:** Characteristics of all the studies included in the meta-analysis.

**Author**	**Year**	**Age (years)**	**Gender (male/female)**	**Liver cirrhosis (numbers)**	**HBV/HCV status (numbers)**	**Number of tumor (solitary/multiple)**	**AFP (ng/ml)**	**Tumor size (cm)**
Belli	2011	63.2	38/27	65	NA	53/12	56 cases ≤ 400, 9 cases>400	3.8
Chang	2012	59.3	403/75	NA	313/77	171/307	110	7.5
Chen	2003	196 cases ≤ 65, 46 cases>65	186/56	0	172/NA	161/81	58 cases ≤ 20, 184 cases>20	19 cases ≤ 2, 223 cases>2
Chen	2015	155 cases ≤ 60, 41 cases>60	156/40	124	178/NA	111/85	94 cases ≤ 200, 102 cases>200	50 cases ≤ 5, 146 cases>5
Chen	2021	332 cases ≤ 60, 82 cases>60	340/74	288	355/NA	362/52	295 cases ≤ 400, 119 cases>400	271 cases ≤ 5, 143 cases>5
Dong	2016	55.2	486/100	536	504/16	586/0	305 cases ≤ 20, 281 cases>20	408 cases ≤ 5, 178 cases>5
Han	2019	NA	394/55	300	415/11	NA	110 cases ≤ 400, 339 cases>400	321 cases ≤ 5, 128 cases>5
Hirokawa	2014	66	17/3	6	17/10	20/0	121	4.6
Hsiao	2017	NA	177/44	86	108/63	117/104	NA	101 cases ≤ 5, 120 cases>5
Huang	2013	946 cases ≤ 65, 94 cases>65	914/126	NA	1040/NA	NA	453 cases ≤ 100, 587 cases>100	629 cases ≤ 5, 411 cases>5
Huang	2015	102 cases ≤ 56, 128 cases>56	173/57	99	152/59	NA	19.8	190 cases ≤ 5, 40 cases>5
Laurent	2005	64	89/19	0	9/12	NA	65 cases ≤ 10, 37 cases>10	NA
Lee	1996	55	42/6	40	NA	NA	9 cases ≤ 20, 39 cases>20	3.3
Lee	2007	47	77/23	NA	83/NA	80/20	59 cases ≤ 1000, 41 cases>1000	13.3
Lee	2012	205 cases ≤ 65, 93 cases>65	222/76	200	146/90	209/86	240 cases ≤ 400, 53 cases>400	NA
Lee	2018	58.4	326/93	249	302/28	376/43	NA	NA
Lee	2019	56.4	428/106	235	280/128	NA	354 cases ≤ 200, 140 cases>200	4.8
Lise	1998	60.2	86/14	78	NA	NA	37 cases ≤ 10, 58 cases>10	5
Liu	2016	54	189/34	199	174/2	168/55	NA	86 cases ≤ 5, 137 cases>5
Liu	2020	NA	208/32	174	183/NA	205/35	137 cases ≤ 20, 107 cases>20	101 cases ≤ 5, 139 cases>5
Park	2018	59	75/17	NA	51/6	69/23	0.103	2.5
Poon	2000	NA	238/50	133	232/NA	NA	NA	124 cases ≤ 5, 164 cases>5
Sasaki	2006	298 cases ≤ 65, 119 cases>65	317/100	272	66/351	318/99	245 cases ≤ 100, 172 cases>100	256 cases ≤ 3, 161 cases>3
Shi	2019	145 cases ≤ 60, 131 cases>60	238/38	140	249/NA	NA	175 cases ≤ 400, 101 cases>400	46 cases ≤ 3, 230 cases>3
Shimada	2008	63	87/30	54	23/78	86/31	23	2.5
Shin	2018	56.4	92/24	82	81/12	116/0	11.9	2.3
Su	2021	59.1	112/47	85	87/47	159/0	11.8	1.58
Takano	2000	60.8	235/65	NA	55/235	265/35	1.616	83 cases ≤ 5, 217 cases>5
Torii	1993	57.7	48/11	56	NA	59/0	NA	30 cases ≤ 2, 29 cases>2
Tsilimigras	2020	66	299/185	148	93/117	NA	8	4.3
Wang	2010	50	380/58	NA	NA	374/54	NA	108 cases ≤ 5, 272 cases>5
Yang	2014	NA	877/208	NA	210/NA	NA	NA	NA
Zeng	2020	36	615/84	355	699/NA	565/134	141 cases ≤ 10, 558 cases>10	5.8
Zhang	2014	48.9	253/49	253	302/NA	238/64	90 cases ≤ 20, 212 cases>20	120 cases ≤ 5, 182 cases>5
Zhang	2021	53.8	357/68	260	376/2	354/71	54.4	3.5
Zhou	2020	NA	278/31	170	274/NA	228/81	203 cases ≤ 400, 106 cases>400	NA
Zhou	2021	683 cases ≤ 60, 134 cases>60	695/122	360	713/NA	NA	452 cases ≤ 400, 365 cases>400	272 cases ≤ 5, 545 cases>5

*NA, not available; HBV, hepatitis B virus; HCV, hepatitis C virus*.

### Correlation Between Surgical Margin and OS

**Figure 2:**
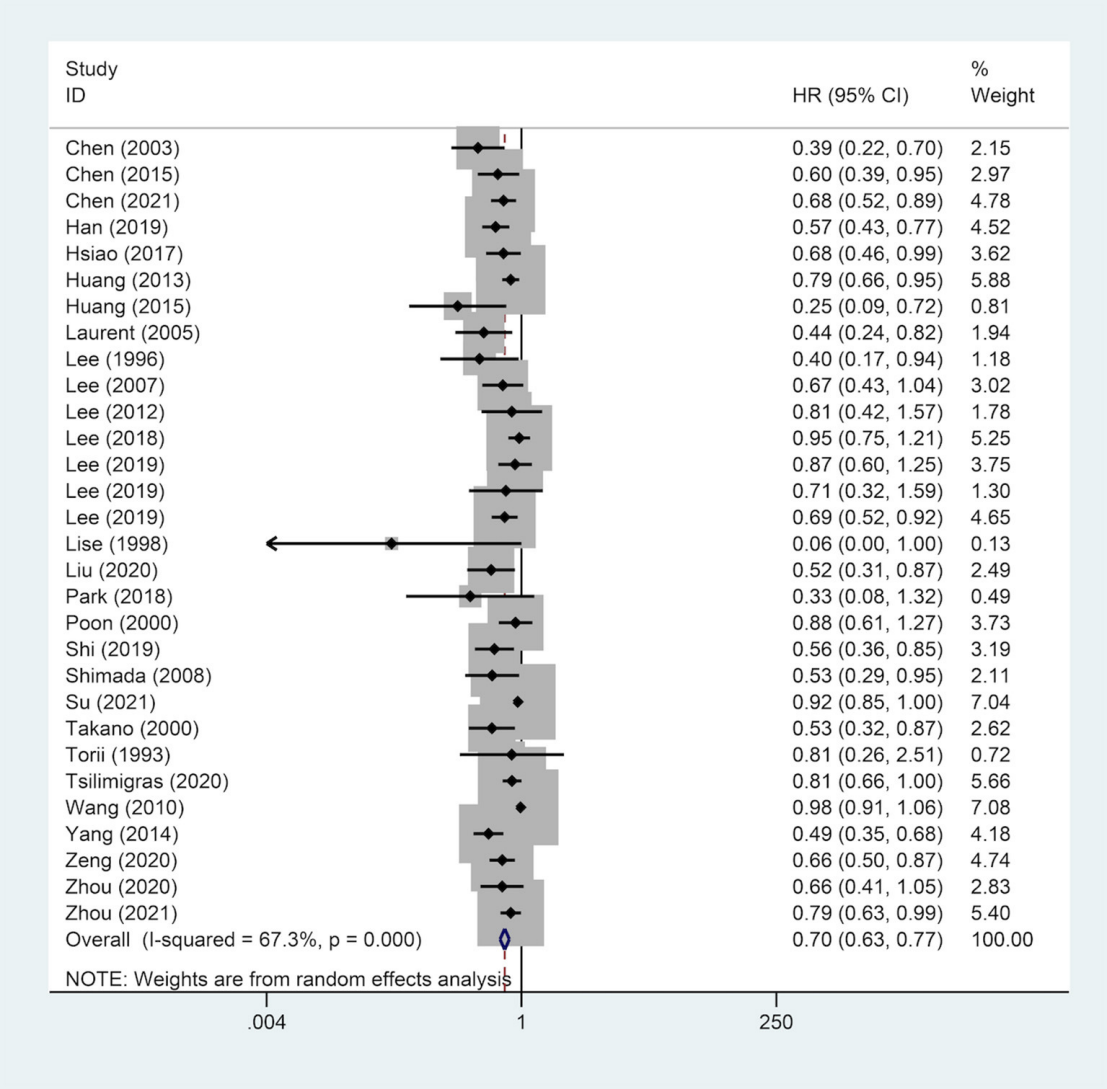
Forest plot of OS of HCC patients receiving wide surgical margin.

A total of 28 studies reported on OS outcomes and pooling analysis of these data revealed that a wide surgical margin is associated with better OS (HR, 0.70; 95% CI, 0.63–0.77) compared to a narrow surgical margin (Figure 2). Subgroups analyses were conducted to explore the potential factors that might affect the impact of the surgical margin on the prognosis (Table 2). This was based on the reported median follow-up time. The studies were divided into 3-year OS and 5-year OS subgroups. The result showed that patients who received a wide resection margin had better mid-and long-term prognosis than those who received a narrow resection margin. Moreover, the gender factor in the subgroups was analyzed and the findings revealed that a narrow surgical margin was a risk factor for OS of patients regardless of men and women. For patients from China or Non-Chinese Asian countries, a wide resection margin was associated with better OS than a narrow resection margin. However, a pooled analysis of three studies from western countries showed that margin width was not associated with prognosis. Additionally, the wide surgical margin group obtained greater OS than that of the narrow surgical margin group in subgroups of hepatitis B surface antigen status (HBsAg) positive/negative and single/multiple tumors.

**Table 2:** Subgroup analysis of the resection margin on the prognosis of patients with HCC.

	**Overall survival (OS)**			**Disease-free survival (DFS)**		
	**No. of studies**	**HR**	**95%CI**	**No. of studies**	**HR**	**95%CI**
3-year survival	5	0.67	0.54–0.82	8	0.57	0.48–0.67
5-year survival	23	0.70	0.63–0.79	19	0.70	0.65–0.76
Male	18	0.68	0.59–0.78	18	0.66	0.60–0.72
Female	9	0.75	0.64–0.89	9	0.66	0.55–0.78
China	19	0.70	0.62–0.78	17	0.67	0.62–0.72
Non-Chinese Asian countries	6	0.68	0.51–0.91	4	0.64	0.46–0.88
Western countries	3	0.54	0.26–1.12	4	0.45	0.30–0.66
HBsAg positive	10	0.71	0.65–0.78	11	0.64	0.57–0.72
HBsAg negative	14	0.66	0.57–0.78	14	0.70	0.64–0.77
Single tumor	9	0.80	0.71–0.92	10	0.67	0.59–0.77
Multiple tumors	7	0.60	0.49–0.73	7	0.66	0.57–0.78
Liver cirrhosis	-	-	-	4	0.71	0.60–0.84
Non-liver cirrhosis	-	-	-	18	0.64	0.58–0.71

*HBsAg, hepatitis B surface antigen; HCC, hepatocellular carcinoma; HR, hazard ratio; CI, confidence interval*.

### Correlation Between Surgical Margin and DFS

**Figure 3:**
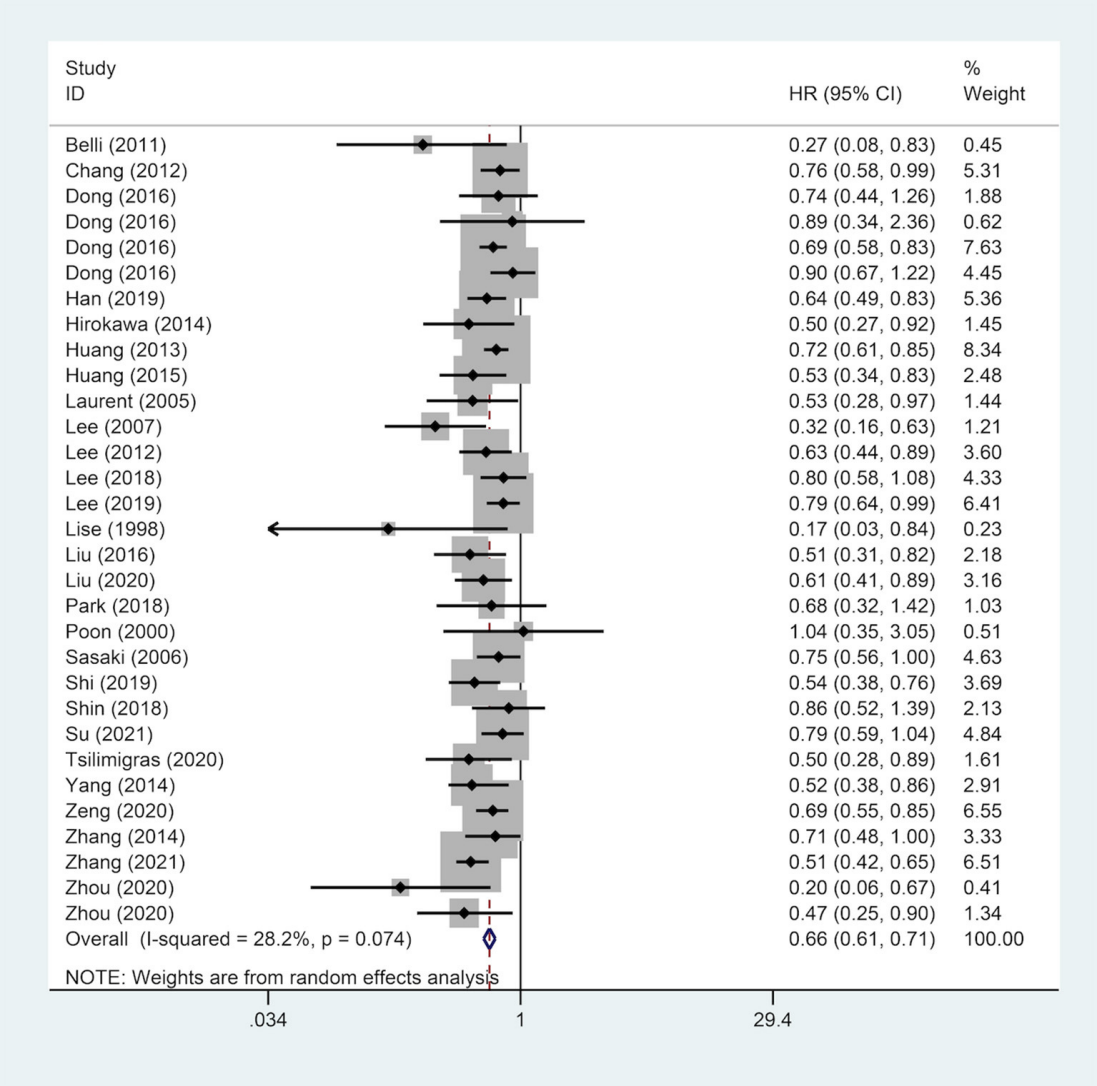
Forest plot of DFS of HCC patients receiving wide surgical margin.

A pooled analysis of DFS data from 27 studies including 9,443 patients revealed that a wide surgical margin was related to better DFS (HR, 0.66; 95% CI, 0.61–0.71) (Figure 3). Further, subgroup analyses were performed based on reported median follow-up time (3-year DFS/5-year DFS), gender (male/female), country (China/Non-Chinese Asian countries/Western countries), HBsAg status (positive/negative), tumor number (single/multiple), liver cirrhosis (patients with/without). As a consequence, a wide surgical margin provided patients with better DFS compared to a narrow surgical margin (Table 2).

### Sensitivity Analysis

After careful selection of studies in sequence, sensitivity analysis outcomes confirmed the excellent robustness of the conclusion that a wide surgical margin could benefit the OS and DFS of patients (Supplementary Figures 1, 2).

## Discussion

The findings of this meta-analysis revealed that surgical margins correlate with the prognosis of HCC patients; besides, a wide surgical margin (≥1 cm) could improve long-term prognosis compared to a narrow surgical margin (<1 cm). This is in line with the results reported in previous articles (39, 40). Through subgroups analyses, we found that the above outcome showed a similar phenomenon in different subgroups except for studies from Western countries. In this analysis, a wide surgical margin did not prolong the OS of patients compared to a narrow surgical margin. This is potentially attributed to the inclusion of a small number of studies (five articles).

No consensus has been reached in academia on whether gender is an independent risk factor for the prognosis of HCC patients after hepatectomy (45). Although there is no direct evidence that gender is a risk factor for HCC prognosis, men have higher smoking rates, alcohol consumption rates, and tumor burden than women (46). A different study found that women have a better long-term prognosis than men, but without statistical difference among patients with HCC lesions maximum size <3 cm or with solitary HCC (47).

Notably, regional factors were also considered in subgroup analysis. The etiology of HCC in different regions is remarkably different. Asian countries, specifically East Asia are dominated by viral hepatitis, whereas HCC etiology in Western countries is mostly related to alcohol (48). Subgroup analyses revealed that despite HCC patients with/without hepatitis B virus (HBV) and liver cirrhosis, a wide surgical margin prolonged the prognosis of patients than a narrow surgical margin. HBV-liver cirrhosis-HCC progression is a vital approach for HCC occurrence. High HBsAg level, lack of antiviral treatment, severe liver cirrhosis are risk factors affecting this process (49–51). However, in single or multiple HCC populations, the wide surgical margin group still yields a better prognosis than the narrow surgical margin group. Nevertheless, a study on a patients with solitary HCC lesions revealed that a wide surgical margin was not a prognostic factor. However, after propensity score matching (PSM), a wide surgical margin still prolongs the prognosis of patients (44). This is possibly because PSM could reduce the confounding bias of OBS and improve the research efficacy by omitting the unmatched study subjects.

Microvascular invasion (MVI) is the presence of tumor emboli in vascular spaces rowed by endothelial cells from the tumor capsule into the liver parenchyma (either hepatic vein or portal vein branches) (52). Based on the distribution and number of MVI, MVI is classified into the following grades, M0: no MVI; M1 (low risk): MVI <5 and the distance from adjacent liver tissues ≤ 1 cm; and M2 (high risk): MVI >5 or the distance from adjacent liver tissues >1 cm (53). Researchers attempted to develop a preoperative model integrating laboratory examinations and imaging examinations to predict MVI. However, its accuracy requires additional validation by large-scale prospective multi-center studies (54). At present, MVI can only be diagnosed by postoperative histopathological examination; this significantly limits the application of MVI in guiding diagnosis and treatment. From MVI to macrovascular invasion, the malignant degree of HCC cells gradually increases and destroys the surrounding tissues; the chance of radical surgery is lost if a macrovascular invasion is formed (55). Therefore, effective surgical plans and postoperative adjuvant treatment can be adopted if timely interventions are implemented at the MVI stage of HCC. This thus minimizes metastasis and HCC recurrence as well as significantly improves the prognosis of patients.

To survive and metastasize, cancer cells must evade the immune system. After cancer cells invade the bloodstream, the classic hematological mechanism believes that platelets, leukocytes, and endothelial cells mediate the related process of metastasis and recurrence (56). New research indicates that MVI provides another path for HCC recurrence and metastasis; besides, HCC cell clusters obtain endothelial coating by protruding into the vessels. This enables evasion of the immune surveillance mechanism and thereby preventing the activation of the coagulation cascade (57–60). Thus, if a liver resection with a narrower surgical margin is performed on patients, theoretically, the residual micrometastasis increases the risk of recurrence (37). Besides, 90% of MVI occurs in the range narrower than 1 cm from the edge of the tumor. If a wider margin is achieved, the incidence of MVI can be reduced, hence significantly preventing tumor recurrence and metastasis (61). However, due to data unavailability, we were unable to analyze the influence of MVI on the results in subgroup analysis. On the other hand, the liver status may be another mechanism of the prognostic influence of the resection margin. Patients who received a wide resection margin tend to have better liver reserves than patients who received a narrow resection margin. Therefore, compared with the narrow surgical margin group, the wide surgical margin group could achieve better OS and DFS.

The surgical margin should however not be blindly enlarged for preventing the recurrence and metastasis of HCC after surgery. Because of the excessively wide surgical margin, more normal liver parenchyma will be removed, causing serious postoperative complications including liver failure, and eventually death (8, 9, 11, 12). Poon et al. (12) revealed that the relatively healthy liver parenchyma should not be sacrificed for obtaining the wider margin, particularly in cirrhotic patients with limited hepatic functional reserves. Another study (25) showed that a wide surgical margin did not improve the OS of patients compared to a narrow surgical margin. This was because of different baselines of the study group and the control group. This was largely reflected in liver cirrhosis, large and multiple tumors.

Previous research evaluated the relationship between surgical margins and prognosis by systematic review and meta-analysis (62, 63). The findings (62) are inconsistent with this meta-analysis and suggests that prognostic benefits are not achieved in patients receiving a resection margin≥1 cm. A small number of articles (5 articles) included a potential reason. The study by Zhong et al. (63) lacked sensitivity analysis, therefore, the reliability and stability of its findings are uncertain. Yet, its results were consistent with this paper's findings. However, it had its limitations. Primarily, although the number of included studies is more than that of previous studies, it is still a relatively small amount when compared to the number of studies in our article (37 articles vs. 7 articles). Besides, subgroup analysis was not performed by Zhong et al. (63). It, therefore, remains unknown whether the conclusion (the prognostic benefit of a wide surgical margin) is affected by other factors.

Our study has worth-mentioning limitations. Firstly, because of the limited number of related studies, comprehensive analysis of different resection margin width could not be performed. Secondly, the study population is from Asia, therefore the results cannot be directly applied to the population in Western countries. Thirdly, most of the included literature is retrospective, thereby hinting a possibility of the potential risk of information bias. Fourthly, because of the non-availability of relevant data, we were unable to perform additional subgroup analyses including MVI and kind of resection (anatomical vs. non-anatomical).

## Conclusion

In conclusion, our meta-analysis revealed that a wide surgical margin (≥1 cm) potentially prolongs the long-term prognosis of HCC patients than a narrow surgical margin (<1 cm). This meta-analysis conducted various subgroup analyses, and the results remained consistent across most factors of median follow-up time, gender, country, hepatitis B surface antigen status, tumor number, and liver cirrhosis.

## Data Availability Statement

The original contributions presented in the study are included in the article/Supplementary Material, further inquiries can be directed to the corresponding author.

## Author Contributions

JZ designed the research process. JX and YL searched the database for corresponding articles and drafted the meta-analysis. JH extracted useful information from the articles above. YS used statistical software for analysis. YH polished this article. All authors had read and approved the manuscript and ensured that this was the case.

## Conflict of Interest

The authors declare that the research was conducted in the absence of any commercial or financial relationships that could be construed as a potential conflict of interest.

## Publisher's Note

All claims expressed in this article are solely those of the authors and do not necessarily represent those of their affiliated organizations, or those of the publisher, the editors and the reviewers. Any product that may be evaluated in this article, or claim that may be made by its manufacturer, is not guaranteed or endorsed by the publisher.

## Supplementary Material

Supplementary Figure 1 Sensitivity analysis of OS for HCC patients receiving wide surgical margin.

Supplementary Figure 2 Sensitivity analysis of DFS for HCC patients receiving wide surgical margin.

Supplementary Table 1 Quality assessment of studies included.

## Abbreviations

HCC: hepatocellular carcinoma
OS: overall survival
DFS: disease-free survival
HR: hazard ratio
CI: confidence interval
HBsAg: hepatitis B surface antigen
OBS: observational study
AFP: alpha-fetoprotein
NOS: Newcastle-Ottawa Scale
HBV: hepatitis B virus
PSM: propensity score matching
MVI: microvascular invasion.
