# Supplementary figures and images for: Prognostic Impact of Surgical Margin in Hepatectomy on Patients With Hepatocellular Carcinoma: A Meta-Analysis of Observational Studies
Source: Front Surg. 2022 Feb 9;9:810479. doi: 10.3389/fsurg.2022.810479 (PMC8863846; doi:10.3389/fsurg.2022.810479)

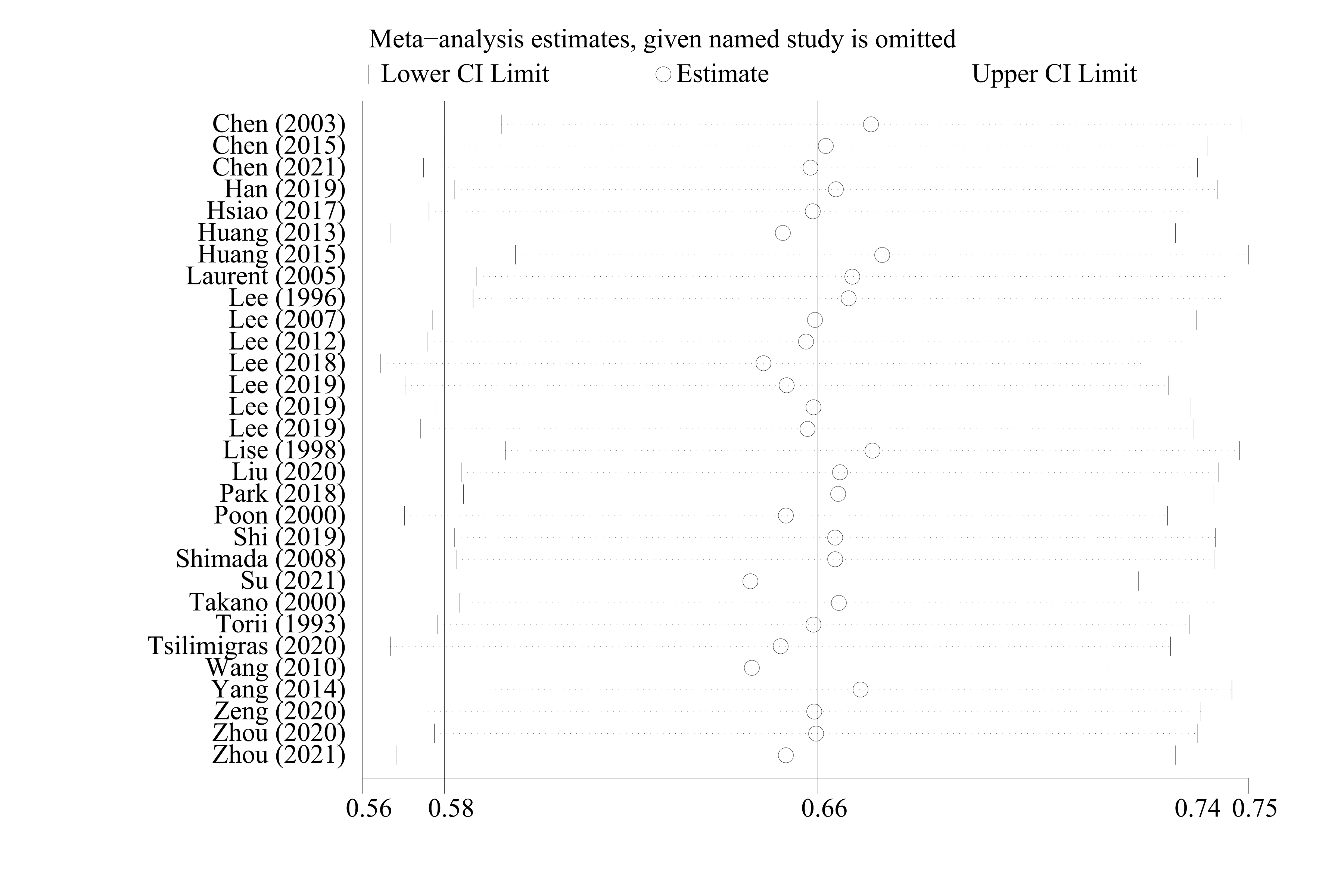

Supplement: Supplementary Figure 1 — Sensitivity analysis of OS for HCC patients receiving wide surgical margin. [file Image_1.TIF]

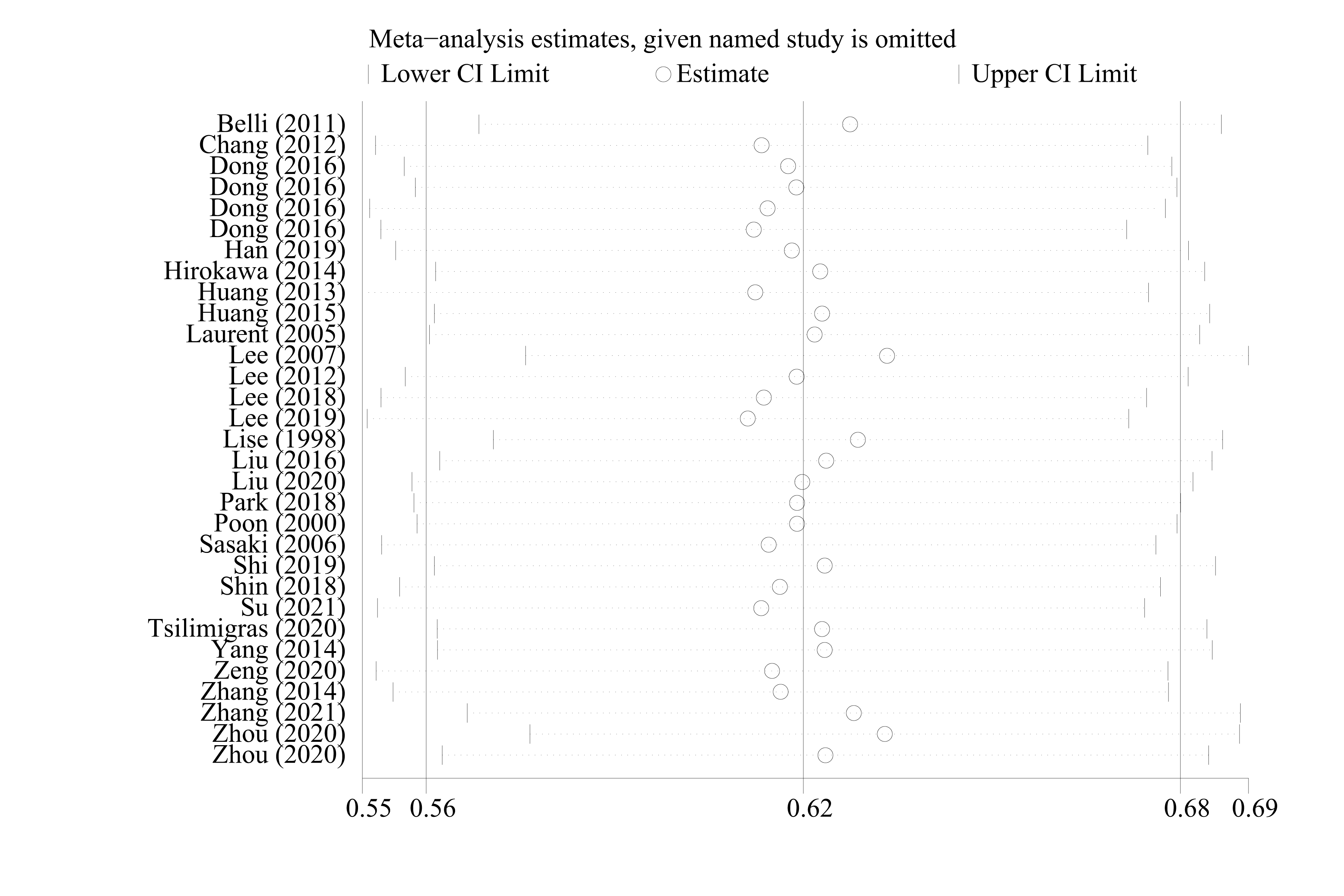

Supplement: Supplementary Figure 2 — Sensitivity analysis of DFS for HCC patients receiving wide surgical margin. [file Image_2.TIF]
