# Supplementary material for: Prognostic Impact of Surgical Margin in Hepatectomy on Patients With Hepatocellular Carcinoma: A Meta-Analysis of Observational Studies
Source: Front Surg. 2022 Feb 9;9:810479. doi: 10.3389/fsurg.2022.810479 (PMC8863846; doi:10.3389/fsurg.2022.810479)
Supplement: Supplementary Table 1 — Quality assessment of studies included. [file Table_1.docx]

**Supplementary Table 1.** Quality assessment of studies included.

| Author, year,  Study (Observational) | **Selection (Out of 4)** | | | | **Comparability**  **(Out of 2)** | **Outcomes (Out of 3)** | | | **Total**  **(Out of 9)** |
| --- | --- | --- | --- | --- | --- | --- | --- | --- | --- |
|  | Representativeness of exposed cohort | Selection of nonexposed cohort | Ascertainment  of exposure | Outcome not present at the start of the study |  | Assessment of outcomes | Length of follow-up | Adequacy of follow-up |  |
| Belli, 2011 | 1 | 1 | 1 | 1 | 1 | 1 | 0 | 1 | 7 |
| Chang, 2012 | 1 | 1 | 1 | 1 | 2 | 1 | 0 | 1 | 8 |
| Chen, 2003 | 1 | 1 | 1 | 1 | 1 | 1 | 0 | 1 | 7 |
| Chen, 2015 | 1 | 1 | 1 | 1 | 1 | 1 | 0 | 0 | 6 |
| Chen, 2021 | 1 | 1 | 1 | 1 | 0 | 1 | 1 | 1 | 7 |
| Dong, 2016 | 1 | 0 | 1 | 1 | 2 | 1 | 1 | 1 | 8 |
| Han, 2019 | 1 | 1 | 1 | 1 | 2 | 1 | 1 | 1 | 9 |
| Hirokawa, 2014 | 1 | 1 | 1 | 1 | 2 | 1 | 1 | 1 | 9 |
| Hsiao, 2017 | 1 | 1 | 1 | 1 | 2 | 1 | 0 | 0 | 7 |
| Huang, 2013 | 1 | 1 | 1 | 1 | 1 | 1 | 1 | 0 | 7 |
| Huang, 2015 | 1 | 1 | 1 | 1 | 2 | 1 | 1 | 0 | 8 |
| Laurent, 2005 | 1 | 1 | 1 | 1 | 2 | 1 | 0 | 1 | 8 |
| Lee, 1996 | 1 | 1 | 1 | 1 | 0 | 1 | 1 | 0 | 6 |
| Lee, 2007 | 1 | 1 | 1 | 1 | 2 | 1 | 1 | 1 | 9 |
| Lee, 2012 | 1 | 0 | 1 | 1 | 1 | 1 | 1 | 1 | 7 |
| Lee, 2018 | 1 | 1 | 1 | 1 | 1 | 1 | 1 | 0 | 7 |
| Lee, 2019 | 1 | 1 | 1 | 1 | 2 | 1 | 1 | 1 | 9 |
| Lise, 1998 | 1 | 1 | 1 | 1 | 1 | 1 | 0 | 1 | 7 |
| Liu, 2016 | 1 | 1 | 1 | 1 | 1 | 1 | 0 | 1 | 7 |
| Liu, 2020 | 1 | 1 | 1 | 1 | 1 | 1 | 1 | 1 | 8 |
| Park, 2018 | 1 | 1 | 1 | 1 | 2 | 1 | 0 | 0 | 7 |
| Poon, 2000 | 1 | 1 | 1 | 1 | 2 | 1 | 0 | 1 | 8 |
| Sasaki, 2006 | 1 | 1 | 1 | 1 | 1 | 1 | 1 | 1 | 8 |
| Shi, 2019 | 1 | 1 | 1 | 1 | 2 | 1 | 1 | 1 | 9 |
| Shimada, 2008 | 1 | 1 | 1 | 1 | 1 | 1 | 1 | 1 | 8 |
| Shin, 2018 | 1 | 1 | 1 | 1 | 2 | 1 | 1 | 1 | 9 |
| Su, 2021 | 1 | 1 | 1 | 1 | 2 | 1 | 1 | 1 | 9 |
| Takano, 2000 | 1 | 1 | 1 | 1 | 1 | 1 | 0 | 0 | 6 |
| Torii, 1993 | 1 | 1 | 1 | 1 | 2 | 1 | 0 | 0 | 7 |
| Tsilimigras, 2020 | 1 | 1 | 1 | 1 | 1 | 1 | 0 | 1 | 7 |
| Wang, 2010 | 1 | 0 | 1 | 1 | 2 | 1 | 0 | 1 | 7 |
| Yang, 2014 | 1 | 1 | 1 | 1 | 2 | 1 | 0 | 0 | 7 |
| Zeng, 2020 | 1 | 1 | 1 | 1 | 2 | 1 | 0 | 1 | 8 |
| Zhang, 2014 | 1 | 1 | 1 | 1 | 2 | 1 | 0 | 1 | 8 |
| Zhang, 2021 | 1 | 1 | 1 | 1 | 2 | 1 | 0 | 1 | 8 |
| Zhou, 2020 | 1 | 1 | 1 | 1 | 1 | 1 | 0 | 1 | 7 |
| Zhou, 2021 | 1 | 1 | 1 | 1 | 2 | 1 | 0 | 1 | 8 |

The observational studies were assessed by the Newcastle-Ottawa Quality Assessment Scale.
